# Non-Chromatographic Purification of Endohedral Metallofullerenes

**DOI:** 10.3390/molecules22050718

**Published:** 2017-04-29

**Authors:** Zhiyong Wang, Haruka Omachi, Hisanori Shinohara

**Affiliations:** 1 Department of Chemistry and Institute for Advanced Research, Nagoya University, Nagoya 464-8602, Japan; zhiyongwang@ruc.edu.cn (Z.W.); omachi@chem.nagoya-u.ac.jp (H.O.); 2 Department of Chemistry, Renmin University of China, Beijing 100872, China

**Keywords:** fullerene, metallofullerene, lewis acid, complexation, cycloparapheneylene

## Abstract

The purification of endohedral metallofullerenes by high performance liquid chromatography is very time-consuming and expensive. A number of rapid and inexpensive non-chromatographic methods have thus been developed for large-scale purification of metallofullerenes. In this review, we summarize recent advances in non-chromatographic purification methods of metallofullerenes. Lewis acid-based complexation is one of the most efficient and powerful methods for separation of metallofullerenes from empty fullerenes. The first oxidation potential of metallofullerenes is a critical factor that affects the separation efficiency of the Lewis acid-based method. Supramolecular methods are effective for separation of fullerenes and metallofullerenes that are different in size and shape. Chemical/electrochemical reduction and exohedral functionalization are also utilized to separate and purify metallofullerenes on a large scale.

## 1. Introduction

Fullerenes are molecular carbon allotropes with closed cage-like structures. The internal hollow space of fullerenes can adopt atoms, ions, clusters, or molecules to form endohedral fullerenes [1,2]. Fullerenes encapsulating one metal atom, i.e., monometallofullerenes M@C_2n_, mainly involve rare-earth metals (e.g., Sc, Y, La, Ce, Gd, Lu) and a number of main-group metals such as Li, Ca, etc. [2,3]. Besides the monometallofullerenes, fullerenes can also accommodate two or three metal atoms, forming dimetallofullerenes M_2_@C_2n_ [4], or trimetallofullerenes M_3_@C_2n_ [5,6]. Other types of metallofullerenes include metallic nitride, carbide, oxide, or sulfide endohedral clusterfullerenes [7,8,9,10,11]. When metal atoms or metallic clusters are entrapped inside fullerenes, electron transfer occurs from the metal atoms or metallic clusters to the fullerene cages. As a consequence, metallofullerenes usually exhibit different chemical and physical properties from the parent empty fullerenes.

Metallofullerenes are attractive materials for a wide range of potential applications. In 2001, Gd-fullerenol (Gd@C_82_(OH)_n_) was found to have a much higher proton relaxivity than that of the commercial magnetic resonance imaging (MRI) contrast agents [12]. Since then, much work has been done for the development of a metallofullerene-based new class of MRI contrast agents [13,14]. Gd-based metallofullerenes also have the potential for cancer therapy. Aggregated Gd@C_82_(OH)_22_ nanoparticles were found to exhibit a high anticancer efficiency and a low toxicity at the same time [15]. It has been found that Gd@C_82_ dissolved in water with the aid of poly(ethylene glycol)-block-poly(2-(*N*,*N*-diethylamino)ethyl methacrylate) can be used for neutron capture therapy [16]. Besides the applications in biomedicine, metallofullerenes also hold great promise in other fields such as organic photovoltaics [17].

Large-scale production and purification of metallofullerenes is a crucial step toward their practical applications. Metallofullerenes are usually produced by an arc-discharge method in the laboratory [2,3]. The yields of metallofullerenes are typically in the milligram scale or even much lower. Further studies on their applications are hampered by their low yields. In recent years, new techniques in the mass production of metallofullerenes have been developed [18]. Some specific metallofullerenes, e.g., Gd@C_82_, can be produced and purified in a gram scale. This progress will pave the way for future research on applications of metallofullerenes.

In most of the previous studies, metallofullerenes were purified by high performance liquid chromatography (HPLC) [2,3]. Solvent-extract of arc-produced soot normally contains a large amount of the so-called empty fullerenes and various types of endohedral fullerenes. Multi-stage HPLC separation on different kinds of chromatographic columns as stationary phases is required to separate/purify the metallofullerenes. In some cases, tedious HPLC recycle performance is necessary to separate metallofullerene fractions with very close retention times [3]. As for large-scale separation/purification of metallofullerenes, the HPLC method is no doubt unfavorable because it is very time-consuming and expensive. Therefore, rapid and inexpensive non-chromatographic methods for separation/purification of metallofullerenes are highly sought after. This review paper summarizes recent advances of non-chromatographic separation/purification of metallofullerenes, which are much more convenient and rapid than the conventional HPLC isolation. In principle, most of the non-chromatographic methods were developed on the basis of the different properties of fullerenes and metallofullerenes toward complexation, oxidation, reduction, or other chemical reactions. The development of non-chromatographic methods makes it much easier to obtain a large quantity of metallofullerenes than it was before.

## 2. Separation/Purification of Metallofullerenes with Lewis Acids

Fullerenes and endohedral fullerenes have π-electron-rich surfaces, so generally they are reactive toward strong Lewis acids. When fullerenes/endohedral fullerenes are mixed with Lewis acids in a solution, complexation and/or redox reaction may take place, leading to precipitation of fullerenes/endohedral fullerenes. The reactivity of fullerenes/endohedral fullerenes toward Lewis acids is correlated to their intrinsic electronic structures. In 1994, Olah and coworkers reported that C_70_ forms a complex with AlCl_3_ in carbon disulfide much faster than C_60_ does [19]. This difference in the reaction rate allows the purification of C_60_ on a large scale. Most of C_70_ can be removed after a reaction period of several days. AlCl_3_ was also used as a strong oxidant to oxidize insoluble metallofullerenes such as Gd@C_72_ and Gd@C_74_ into soluble cations [20,21]. In 2009, Stevenson and coworkers reported that metallic nitride and oxometallic endohedral fullerenes exhibit different reactivity toward Lewis acids such as AlCl_3_ or FeCl_3_ [22]. They found that the reactivity order was Sc_4_O_2_@*I_h_*-C_80_ > Sc_3_N@C_78_ > Sc_3_N@C_68_ > Sc_3_N@*D*_5*h*_-C_80_ > Sc_3_N@*I_h_*-C_80_. The empty-cage fullerenes, such as C_60_ and C_70_, are largely unreactive toward the Lewis acids. A separation scheme was designed for the separation of the metallofullerenes on the basis of selective complexation and precipitation.

**Figure 1:**
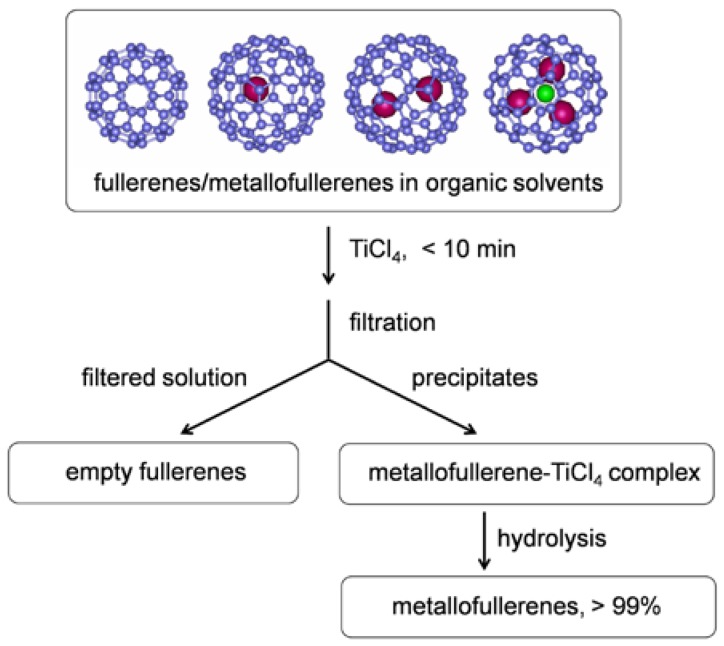
Separation and purification scheme of metallofullerenes on the basis of selective complexation with TiCl_4_.

Recently, our group and coworkers reported a TiCl_4_-based non-chromatographic purification method [23,24], as schematically described in Figure 1, which is much more effective and easy-to-handle than any other Lewis acids. We found that metallofullerenes and empty fullerenes behave very differently toward TiCl_4_ complexation. When a certain amount of TiCl_4_ is added to an organic solution of fullerene/metallofullerene mixture, the metallofullerenes form a complex with TiCl_4_ immediately and then precipitate from the solution. The empty fullerenes, on the contrary, are almost unreactive toward TiCl_4_ and remain in the solution. The metallofullerenes and empty fullerenes can be separated from each other very easily by filtration. Then, the metallofullerenes are recovered completely by hydrolysis of the metallofullerene-TiCl_4_ complex. One of the advantages of this TiCl_4_-based method is that the purification process is very fast. Typically, only several minutes is needed to precipitate the metallofullerenes completely.

**Figure 2:**
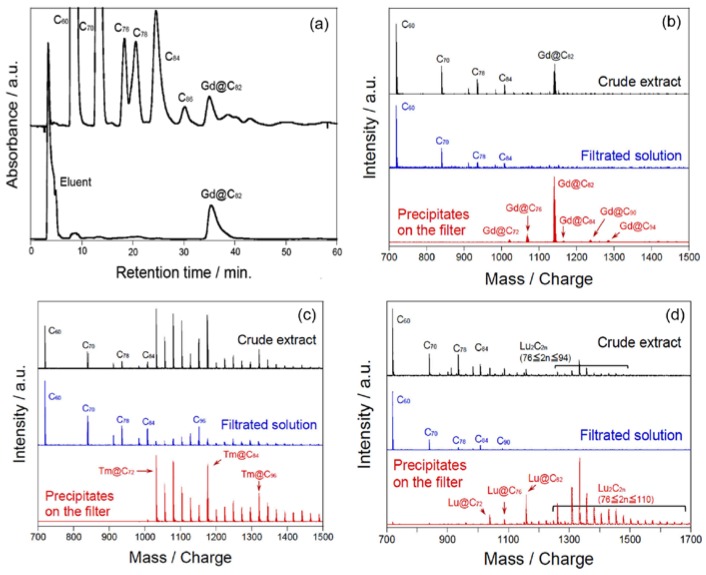
(**a**) HPLC profiles of crude extract containing Gd@C_82_ and empty fullerenes (top line) and Gd@C_82_ purified by complexation with TiCl_4_ (bottom line); (**b**–**d**) Positive-ion laser desorption mass spectra (LD-MS) of crude extracts, filtered solutions, and precipitates for Gd-, Tm-, and Lu-metallofullerenes, respectively. Empty fullerenes are removed completely after a single step treatment with TiCl_4_. Reproduced with permission from [23]. Copyright 2012 American Chemical Society.

The TiCl_4_-based method is applicable to the separation/purification of all of the metallofullerenes that we have examined so far. Monometallofullerenes M@C_2n_ based on both trivalent metal ions (e.g., Y^3+^, La^3+^, Ce^3+^, Er^3+^, Gd^3+^, Lu^3+^) and divalent metal ions (e.g., Sm^2+^, Eu^2+^, Tm^2+^, Yb^2+^) can be separated from empty fullerenes with up to >99% purity in a single step through complexation with TiCl_4_. Additionally, dimetallofullerenes M_2_@C_2n_, and clusterfullerenes, e.g., M_2_C_2_@C_2n_ and M_3_N@C_2n_, can also be purified with TiCl_4_ treatment. Some typical results of separation/purification with TiCl_4_ are described in Figure 2.

**Figure 3:**
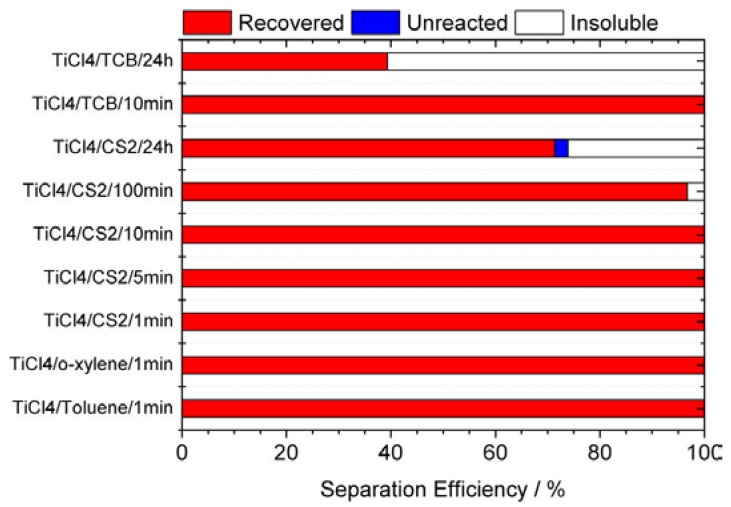
Separation efficiency of La@C_82_ with TiCl_4_ in solvents of carbon disulfide, toluene, *o*-xylene, and 1,2,4-trichlorobenzene (TCB) for various reaction periods. Reproduced with permission from [23]. Copyright 2012 American Chemical Society.

We have examined the effects of solvent and reaction time on the separation efficiency of the La@C_82_ metallofullerene (Figure 3). When the reaction time is within 10 min, a separation efficiency of >99% is achieved for solvents of carbon disulfide, toluene, *o*-xylene, or 1,2,4-trichlorobenzene (TCB). However, the separation efficiency decreases gradually if the reaction time is longer than 100 min, which is presumably caused by an irreversible evolution of the La@C_82_-TiCl_4_ complex under ambient conditions.

**Figure 4:**
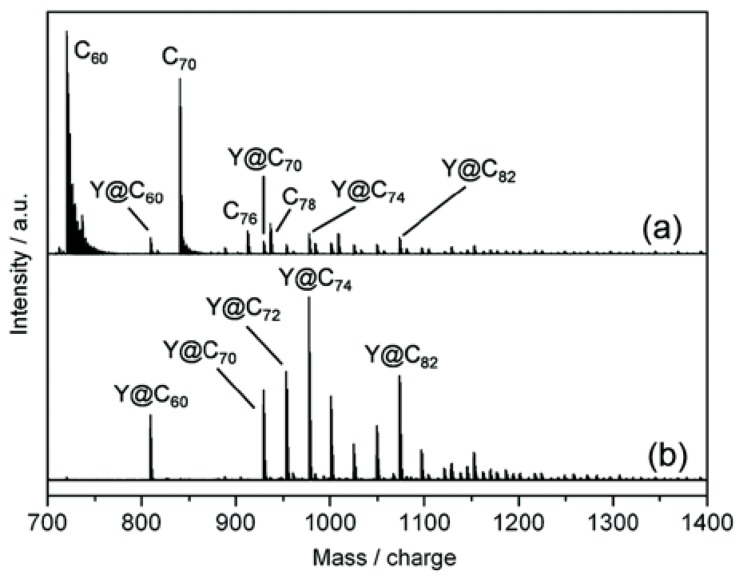
Positive-ion LD-MS of (**a**) crude extract of yttrium soot and (**b**) Y-metallofullerene derivatives separated with TiCl_4_. The derivatives Y@C_2n_(CF_3_)_m_ are decomposed by laser irradiation, so the fragment signals from Y@C_2n_^+^ are observed in the spectra. Reprinted from [25] with permission from Wiley-VCH Verlag GmbH & Co. KGaA, Weinheim.

Importantly, in addition to pristine metallofullerenes, TiCl_4_ is equally effective at separating/purifying metallofullerene derivatives. Recently, we reported the solvent-extraction and isolation of missing small-bandgap metallofullerenes through exterior functionalization with CF_3_ groups [25,26]. The trifluoromethylated derivatives of small-bandgap metallofullerenes, Y@C_2n_(CF_3_)_m_ (2n = 60, 70, 72, or 74, m = 1 or 3), exhibit high reactivity toward TiCl_4_ (Figure 4) [25], which are similar to the case of pristine metallofullerenes. The removal of the empty fullerenes (e.g., C_60_, C_70_) with TiCl_4_ is a critical step for final purification of M@C_2n_(CF_3_)_m_. Because the derivatives have very similar retention times with C_60_ and C_70_ on chromatographic columns, it would be very difficult to isolate the derivatives by HPLC if the sample contains large amounts of C_60_ and C_70_.

**Figure 5:**
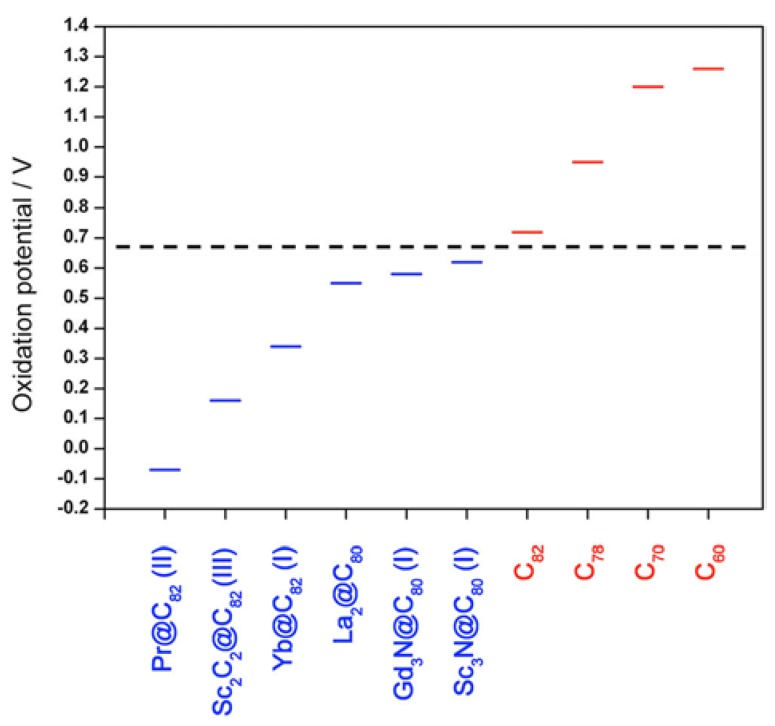
Plot of first oxidation potential (vs. ferrocene (Fc/Fc^+)^) of fullerenes and metallofullerenes and the threshold (dashed line) in the first oxidation potential for reaction with TiCl_4_. Blue and red colors denote the reactive and nonreactive fullerenes/metallofullerenes, respectively. Reproduced with permission from [24]. Copyright 2012 American Chemical Society.

To gain a better understanding on the complexation of metallofullerenes with TiCl_4_, we investigated the origin and mechanism of the separation/purification process. We found that the first oxidation potential is a crucial physical parameter that determines the reactivity of fullerenes and metallofullerenes toward TiCl_4_ complexation [24]. The reactivity of fullerenes and metallofullerenes with first oxidation potentials ranging from −0.07 to 1.26 V versus Fc/Fc^+^ (Fc = ferrocene) has been examined. Interestingly, metallofullerenes with first oxidation potentials of −0.07–0.62 V are reactive toward TiCl_4_; on the other hand, the empty fullerenes with first oxidation potentials of 0.72–1.26 V are almost nonreactive. Thus, the threshold value in the first oxidation potential for reaction with TiCl_4_ is determined to be 0.62–0.72 V (Figure 5). To the best of our knowledge, all of the reported metallofullerenes have first oxidation potentials below or close to this threshold value. Thus, the TiCl_4_-based purification method can be applied to virtually all of the reported metallofullerenes. For example, a nitride clusterfullerene Dy_3_N@*I_h_*-C_80_, which has a first oxidation potential of 0.70 V, can be purified efficiently with TiCl_4_ in our laboratory.

The reaction mechanism between metallofullerenes and TiCl_4_ was investigated by monitoring the absorption spectrum. It was found that the absorption features of metallofullerene disappear and a new band from metallofullerene cation arises immediately after the addition of TiCl_4_ [24]. This dramatic change indicates that electron transfer occurs from metallofullerene to TiCl_4_. Element analysis reveals that the molar ratio of metallofullerene to TiCl_4_ is about 1:18-19 in the metallofullerene-TiCl_4_ complex [23]. The metallofullerene might be surrounded by the TiCl_4_ molecules. Further work is needed to determine the precise structure of the complex. There are several reports on the interaction between TiCl_4_ and empty cage fullerenes [27,28,29]. For example, TiCl_4_ has been used to chlorinate C_70_ fullerene [27]. The structures of TiCl_4_-solvated empty fullerenes, such as C_60_•3TiCl_4_ and C_70_•2TiCl_4_, have been crystallographically characterized [28,29]. It is possible to disclose the structure of the metallofullerene-TiCl_4_ complex by crystallographic study in the future.

Besides TiCl_4_, there are some other metal halides (e.g., AlCl_3_, FeCl_3_, and CuCl_2_) that can be used to separate and purify metallofullerenes through selective complexation [22,30,31,32]. The metal halides differ from each other with respect to their complexation abilities toward metallofullerenes. As compared with TiCl_4_, CuCl_2_ is a much weaker Lewis acid. It has been found that the precipitation threshold in the first oxidation potential for CuCl_2_ is about 0.19 V (vs. Fc/Fc^+^) [30], which is much lower than that for TiCl_4_ (0.62–0.72 V). Therefore, CuCl_2_ can be used to selectively precipitate the most reactive metallofullerenes. For example, metallofullerenes Sc_3_C_2_@*I_h_*-C_80_ and Sc_4_O_2_@*I_h_*-C_80_, with a first oxidation potential of −0.03 and 0.00 V, respectively, can be separated rapidly from a more inert metallofullerene Sc_3_N@*I_h_*-C_80_ by using CuCl_2_; Sc_3_N@*I_h_*-C_80_ can be subsequently purified through a second step of complexation with a stronger Lewis acid (e.g., FeCl_3_) [30]. For a mixture of metallofullerenes, the purification process can be greatly simplified if a number of Lewis acids are used consecutively to separate the metallofullerenes into distinct classes in terms of their reactivity. Stevenson and coworkers investigated the reactivity of a series of Lewis acids and reported the order of increasing reactivity as follows: CaCl_2_ < ZnCl_2_ < NiCl_2_ < MgCl_2_ < MnCl_2_ < CuCl_2_ < WCl_4_ ≪ WCl_6_ < ZrCl_4_ < AlCl_3_ < FeCl_3_ [31]. They have isolated Gd_3_N@*D_2_*(35)-C_88_ and CeLu_2_N@*I_h_*-C_80_ by using the Lewis acid method [32,33]. The precipitation thresholds in the first oxidation potential of metallofullerenes for different Lewis acids are summarized in Table 1.

Because the metallofullerene isomers usually have different oxidation potentials, it is possible to separate them on the basis of selective chemical oxidation. In 2005, Echegoyen and coworkers reported that *I_h_* and *D*_5*h*_ isomers of Sc_3_N@C_80_ can be separated by selective oxidation with tris(*p*-bromophenyl)aminium hexachloroantimonate (TPBAH) [34]. Because of its lower first oxidation potential, the *D*_5*h*_ isomer of Sc_3_N@C_80_ is oxidized preferentially by TPBAH. The resulted cation of Sc_3_N@*D*_5*h*_-C_80_ and neutral Sc_3_N@*I_h_*-C_80_ are then separated with a silica column. This method was recently modified by using acetylferrocenium tetrakis(pentafluorophenyl)borate ([Fe(COCH_3_C_5_H_4_)Cp][TFAB]) instead of TPBAH as the oxidant [35]. The oxidation ability of [Fe(COCH_3_C_5_H_4_)Cp][TFAB] is weaker than that of TPBAH. In addition to Sc_3_N@*D*_5*h*_-C_80_ and Sc_3_N@*I_h_*-C_80_, Sc_3_N@C_68_ and Sc_3_N@C_78_ are also purified or fractionated by using [Fe(COCH_3_C_5_H_4_)Cp][TFAB].

## 3. Size-Selective Separation/Purification of Metallofullerenes with Cycloparaphenelenes

Supramolecular host-guest chemistry is a powerful tool for non-chromatographic fullerene separation [36,37], and there are many examples of the complexation with metallofullerenes [38,39,40,41,42,43]. Akasaka and coworkers reported the selective extraction of lanthanum endohedral metallofullerenes from the as-synthesized mixture by the complexation with azacrown ether, 1,4,7,10,13,16-hexaazacyclooctadecane [38]. However, the selective extraction of a single metallofullerene has not been demonstrated yet due to the difficulty of the size-selective separation.

**Figure 6:**
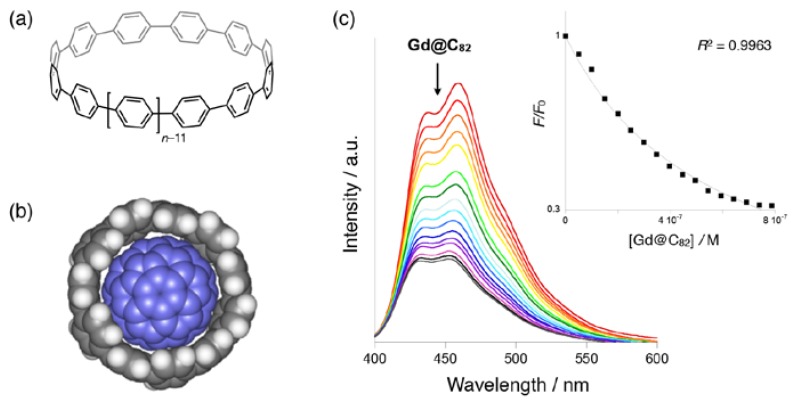
(**a**) Chemical Structure of [*n*]Cycloparaphenylene ([*n*]CPP). (**b**) A space-filling model of C_82_@[11]CPP. (**c**) Fluorescence spectra and plot for determination of the binding constant of Gd@C_82_/[11]CPP titration. Reprinted from [52] with permission from Wiley-VCH Verlag GmbH & Co. KGaA, Weinheim.

For the achievement of size-selective extraction, we have used cycloparapheneylenes (CPPs), which are cyclic compounds composed of solely *para*-substituted benzenes (Figure 6a) [44,45,46]. Some groups including the present laboratory have already reported that the pi-conjugated system is effective for the metallofullerene-selective complexation [40,41,42,43]. In order to realize size-selectivity, cyclic paraphenylene cavities are suitable candidates for the encapsulation of fullerenes [47,48,49]. Notably, [11]CPP has an ideal inner space with a diameter of 1.51 nm [50] for the inclusion of M@C_82_ families (0.85 nm in diameter) considering the interlayer distance of convex–concave pi-pi interactions such as fullerene nano-peapods (ca. 0.33 nm) [51]. A theoretical calculation also suggested that C_82_ could be fit almost completely into the cavity of [11]CPP (Figure 6b) [52].

After the establishment of the first selective synthesis of [11]CPP [53], we determined the binding abilities of [11]CPP to a series of C_82_-based metallofullerenes by fluorescence quenching experiments [52]. The fluorescence intensity of [11]CPP gradually decreased with the addition of metallofullerenes (Figure 6c). The binding constant (*K*_a_) of [11]CPP with Gd@C_82_ is 1.8 × 10^6^ M^−1^, which is one of the highest values for the complexes of higher metallofullerenes [40,41,42]. With other metallofullerenes such as Tm@C_82_, Lu_2_@C_82_, and Sc_3_N@C_80_, subequal binding constants were recorded. In contrast, the strong fluorescence quenching of larger [12]CPP [54] (1.66 nm in diameter) was not observed with the addition of C_82_-based metallofullerenes.

**Figure 7:**
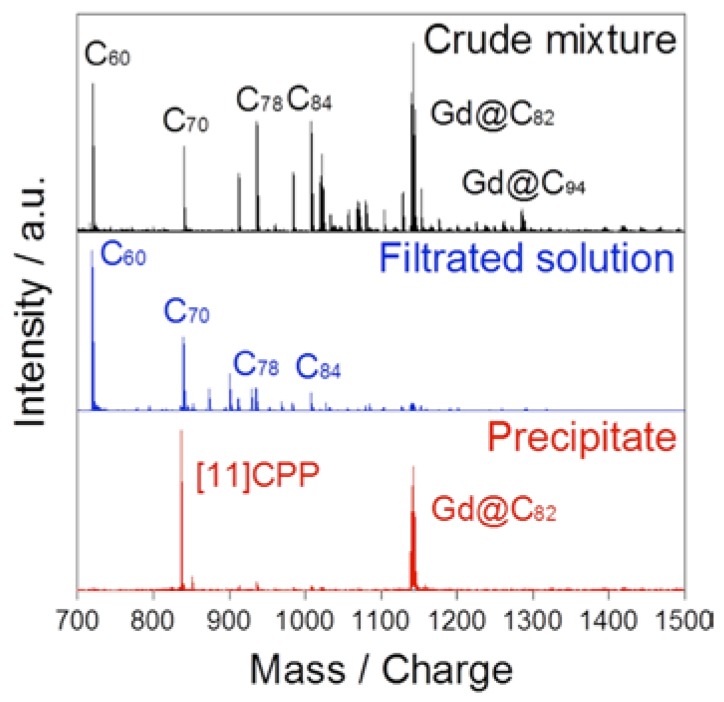
Positive-ion LD-MS spectra of the crude mixture with low contents of metallofullerenes, the filtrate, and the precipitate after the addition of [11]CPP. Reprinted from [52] with permission from Wiley-VCH Verlag GmbH & Co. KGaA, Weinheim.

The separation protocol of Gd@C_82_ is described below. To a toluene solution of the fullerene mixture from raw soot that included Gd@C_82_, [11]CPP was added. After the solution was concentrated under reduced pressure, a brown solid precipitated out. Low solubility of the complex between Gd@C_82_ and [11]CPP realizes this non-chromatographic extraction separation technology. A positive-ion laser desorption mass spectra (LD-MS) of the crude extract, the filtrate solution, and the separated precipitate are shown in Figure 7. The depression of the Gd@C_82_ peak in the filtrate and a strong peak of Gd@C_82_ with the fragmentation of [11]CPP from the precipitate were observed. These results indicate that most of Gd@C_82_ are collected as the precipitate. It should be emphasized that higher metallofullerenes such as Gd@C_94_ are not extracted by the complexation with [11]CPP due to size mismatch between the fullerene molecule and the cavity of [11]CPP.

Table 2 summarizes a comparison of the extraction efficiency of Gd@C_82_ (yield, amount of solvent used, and time required) using the complexation method and the multistage HPLC method. The HPLC extraction of Gd@C_82_ was carried out for the same amount (10 mg) of crude fullerene mixture. The amounts of each Gd@C_82_ obtained were determined from the reported absorption coefficient. Although the HPLC method is somewhat superior in yield (72 μg vs. 56 μg), the present complexation method is much superior to the multistage HPLC method in terms of the amount of solvent used (50 mL vs. 5 L) and the time required for extraction (30 min vs. 5 h).

After our report of the size-selective extraction of metallofullerenes with CPPs, the group of Yamago revealed the partial electron transfer from [11]CPP to La@C_82_ [55]. This polar complex formation might support the selective recognition of metallofullerene and trigger a change to poor solubility in non-polar solvents like toluene.

## 4. Separation/Purification of Metallofullerenes through Chemical/Electrochemical Reduction

The electrochemical properties of empty fullerenes and metallofullerenes have been extensively studied during the past two decades [3,56]. The reported redox potentials provide useful information for separation/purification of metallofullerenes based on selective reduction/oxidation. Due to their higher first reduction potentials, the metallofullerenes are much easier to reduce as compared with the empty fullerenes. For example, La@C_82_ and La_2_@C_80_ in TCB extract of La soot can be selectively reduced into anions electrochemically [57]. The anions can easily be separated from neutral fullerenes as they have different solubilities in organic solvents. Subsequent oxidation of the anions with dichloroacetic acid leads to the recovery of the neutral metallofullerenes. In addition to the classical metallofullerene M@C_82_, there are a large number of the so-called missing small-bandgap metallofullerenes such as M@C_60_ and M@C_70_ [2]. The small-bandgap metallofullerenes are insoluble in organic solvents because they tend to form polymers or oligomers in raw soot. Alford and coworkers reported that the small-bandgap metallofullerenes become soluble and stable in organic solvents after electrochemical reduction [58], thus enabling further separation/purification of these unconventional metallofullerenes.

When an organic solvent such as carbon disulfide or *o*-xylene is used to extract metallofullerenes from raw soot, a large number of empty fullerenes are also dissolved in the solvent. To selectively extract metallofullerenes, some chemical reduction-based routes have been developed. Gu and coworkers reported the simultaneous reduction and extraction of metallofullerenes using Al-Ni alloy in a mixed solvent of toluene and tetrahydrofuran (THF) [59,60]. They found that the composition of the extracted metallofullerenes is dependent on the composition of the solvents used. Specifically, a mixture of Gd@C_82_/Gd_2_@C_80_ or pure Gd_2_@C_80_ can be extracted by tuning the ratio of toluene to THF. Their work provides a simple method for selective extraction of M_2_@C_80_ metallofullerene. Kodama and coworkers reported that Ce-metallofullerenes (Ce@C_82_, Ce_2_@C_78_, Ce_2_@C_80_) can be extracted selectively using a mixed solvent of triethylamine and acetone [61]. They proposed that the reduction of Ce-metallofullerenes by triethylamine or the formation of metallofullerene-triethylamine donor-accepter complexes is responsible for the selective extraction of metallofullerenes.

Unlike non-polar solvents such as carbon disulfide and *o*-xylene, *N*,*N*-dimethylformamide (DMF) was found to be a special solvent that can dissolve metallofullerenes preferentially, excepting empty fullerenes [62,63,64,65,66]. Akasaka and coworkers proposed that the metallofullerenes dissolved in DMF are actually in the form of anions, not neutral metallofullerenes; these might be formed during the high-temperature extraction process [66]. As such, metallofullerenes with high first reduction potentials dominate the DMF extract.

## 5. Separation/Purification of Metallofullerenes through Exohedral Organic Reactions

**Figure 8:**
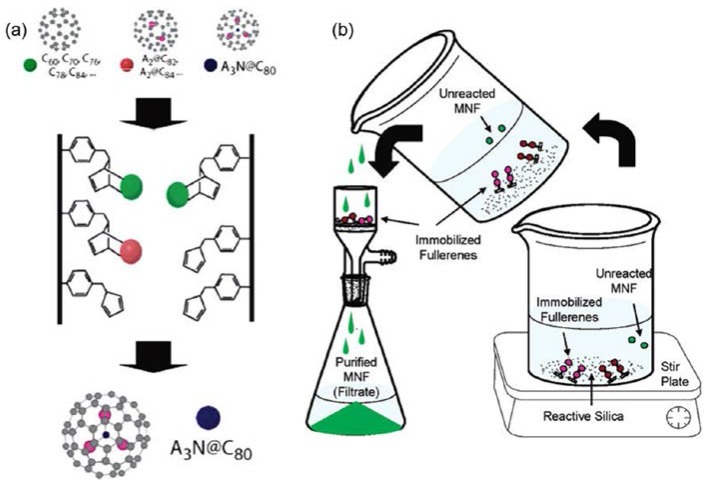
Schematic illustration of purification processes for metal nitride clusterfullerenes. (**a**) The cyclopentadienyl-functionalized resin method. Reproduced with permission from [67]. Copyright 2005 American Chemical Society. (**b**) The “Stir and Filter Approach” using reactive silica (MNF = metallic nitride fullerenes). Reproduced with permission from [68]. Copyright 2006 American Chemical Society.

Organic chemistry of fullerenes/metallofullerenes has been an active research area since their discovery. Exohedral functionalization of fullerenes and metallofullerenes provides new routes to realize efficient separation of fullerenes and metallofullerenes. Dorn and coworkers reported that metal nitride clusterfullerenes can be separated from empty fullerenes and classical metallofullerenes by using a column packed with cyclopentadienyl-functionalized resin (Figure 8a) [67]. Empty fullerenes and metallofullerenes are bound to the resin by the Diels-Alder reaction, whereas, metal nitride clusterfullerenes remain intact and pass through the column due to their inertness. The bound empty fullerenes and metallofullerenes can be recovered upon heating in the presence of maleic anhydride. Stevenson and coworkers reported an alternative way to separate metal nitride clusterfullerenes through selective reaction with cyclopentadienyl- or amino-functionalized silica, which they call the “Stir and Filter Approach” (SAFA) (Figure 8b) [68,69,70,71,72]. This approach can be used to remove Sc_3_N@*D*_5*h*_-C_80_ rapidly from its structural isomer, Sc_3_N@*I_h_*-C_80_ [70]. Further studies demonstrate that the content of water and the substituents on the aromatic solvent molecules have significant effects on the separation efficiency of metal nitride clusterfullerenes when using amino-functionalized silica [72].

In addition to the support-based separation strategies, some support-free chemical methods have also been reported for purification of metal nitride clusterfullerenes. For example, molten 9-methylanthracene was employed to react with a solid mixture of empty fullerenes and metal nitride clusterfullerenes [73]. The empty fullerenes were converted to 9-methylanthracene adducts that have different solubilities in organic solvents as compared with intact metal nitride clusterfullerenes. After washing away the fullerene derivatives with ether, metal nitride clusterfullerenes was enriched to about 60% purity within one day. Another example of a non-chromatographic method is based on selective extraction with 2-aminoethanol [74]. Empty cage fullerenes are much more reactive than the endohedral metallofullerenes Sc_3_N@C_2n_ (n = 34, 39, 40) toward nucleophilic 2-aminoethanol. The empty cage fullerenes were selectively transferred to the 2-aminoethanol layer after reaction, and the endohedral metallofullerenes Sc_3_N@C_2n_ were enriched in the original solution. Wang and coworkers reported a purification method based on Prato-type 1,3-dipolar cycloaddition [75]. Both empty fullerenes and metal nitride clusterfullerenes undergo cycloaddition with *N*-ethylglycine and paraformaldehyde [76]; subsequent retro-cycloaddition using 3-chloroperoxybenzoic acid, however, only takes place for the derivatives of metal nitride clusterfullerenes. This difference realizes a facile separation of metal nitride clusterfullerenes from empty fullerenes.

## 6. Summary

In order to realize convenient and rapid separation/purification of metallofullerenes, a number of non-chromatographic methods have been developed. Lewis acid-based complexation is one of the most efficient and powerful methods for separation of metallofullerenes from empty fullerenes. For this Lewis acid-based method, the separation efficiency is basically determined by the first oxidation potentials of the metallofullerenes. Supramolecular methods are effective for separation of fullerenes and metallofullerenes that are different in size and shape. The metallofullerene M@C_82_ can be isolated through selective complexation with [11]CPP. Chemical/electrochemical reduction and exohedral functionalization are also utilized to separate metallofullerenes in a large scale. It is much easier to obtain a large quantity of metallofullerenes than it was before by using the non-chromatographic methods. The development of non-chromatographic methods is a crucial step toward future industrial production of metallofullerenes.

## Figures and Tables

**Table 1:** The precipitation threshold in the first oxidation potential of metallofullerenes for different Lewis acids.

Lewis Acids	Precipitation Threshold (vs. Fc/Fc^+^)
TiCl_4_	0.62–0.72 V
WCl_6_, ZrCl_4_, AlCl_3_, FeCl_3_	0.6 V
MgCl_2_, MnCl_2_, WCl_4_	0.1–0.5 V
CuCl_2_	0.19 V
CaCl_2_, ZnCl_2_, NiCl_2_	0.1 V

**Table 2:** A comparison of the extraction efficiency between a multistage HPLC method and the cycloparapheneylene (CPP) method from 10 mg of crude extract.

	HPLC Method	CPP Method
Collected Gd@C_82_	72 µg	56 µg
Amount of solvent used	~5 L	~50 mL
Time required	~5 h	~30 min
